# Integrating eye health into a child health policy in Tanzania: global and national influences

**DOI:** 10.1093/heapol/czaf029

**Published:** 2025-06-23

**Authors:** Aeesha Nusrat Jehan Malik, Neil Spicer, Milka Mafwiri, Clare Gilbert, Joanna Schellenberg

**Affiliations:** International Centre of Eye Health, London School of Hygiene and Tropical Medicine, Keppel Street, London WC1E 7HT, United Kingdom; Department of Infectious Disease Epidemiology, London School of Hygiene and Tropical Medicine, Keppel Street, London WC1E 7HT, United Kingdom; Department of Ophthalmology, Muhimbili University of Health and Allied Sciences, Malik Road, Dar es Salaam 65000, United Republic of Tanzania; International Centre of Eye Health, London School of Hygiene and Tropical Medicine, Keppel Street, London WC1E 7HT, United Kingdom; Department of Infectious Disease Epidemiology, London School of Hygiene and Tropical Medicine, Keppel Street, London WC1E 7HT, United Kingdom

**Keywords:** child health, health policy, eye health

## Abstract

Global consensus has shifted to focus on how children can be supported to not only ‘survive’ but to ‘thrive’. Blindness and visual loss in early childhood undermine a child’s ability to thrive, affecting psychomotor, cognitive, and social development leading to life-long consequences for educational attainment, employment, economic and social status, and wellbeing. Despite this, eye health for children under the age of 5 years has been neglected, and not politically prioritized. In Tanzania, policy makers decided in 2019 to include eye conditions in the national Integrated Management of Newborn and Childhood Illness (IMNCI) programme, despite eye health not being part of the global World Health Organization/UNICEF IMNCI strategy. We conducted a qualitative policy analysis to explore enabling factors and barriers to this policy change. The interviews were semi-structured with key actors selected purposively and by snowball sampling, including those with a role in child and eye health at national and global levels. We used an adapted Shiffman and Smith framework (Generation of political priority for global health initiatives: a framework and case study of maternal mortality. *Lancet* 2007;370:1370–9) to guide the interviews and analysis, and the Consolidated Criteria for Reporting Qualitative Research for planning and reporting. This study shows how rapidly one country altered its overall child health policy to include eye health, driven by good quality collaborative research and collective action (cohesive policy community) which importantly included co-design with the decision makers (Ministry of Health actors). These developments coincided with the shift in the international agenda moving from ‘survive to thrive’ in child health which was leveraged to include eye care in the national strategy.

Key messages

- This study represents the first study examining agenda setting for eye health policy integration into national child health policies, utilizing an adapted Shiffman and Smith framework that can be applied in different contexts beyond Tanzania.
- The global shift in child health policy from mere ‘survival’ to helping children ‘thrive’ creates a crucial window for incorporating eye health into the Integrated Management of Newborn and Childhood Illness strategy and other child health policies.
- The Tanzanian experience studied in this paper demonstrates that eye health can gain political priority through leveraging existing policy networks, framing ideas within current child health priorities, and working collaboratively with policy makers to develop local evidence.
- Addressing the ‘policy gap’ in child health requires governments and key stakeholders to work together from evidence-building through implementation to ensure eye health becomes an integral component of child health policies globally.

## Introduction

Blindness and vision loss in early childhood has far reaching consequences for the child, affecting their psychomotor and cognitive development, educational attainment, employment, economic and social status, and wellbeing (Keil *et al.* 2016, Solebo *et al.* 2017). Children who are blind are also more likely to die in childhood than those with good vision, particularly if they live in low-resource settings (WHO 1999). Most blind children are either born with congenital conditions or become blind before the age of 5 years from acquired conditions (Rahi and Gilbert 2022).

Globally, 70.2 million children are estimated to have sight loss, 1.4 million of whom are blind (Burton *et al.* 2021). The majority of children with sight loss, up to 80%, are living in Africa, Asia, and South America, reflecting the higher estimated prevalence and the large child populations in those regions. The available evidence suggests that in all regions, most blindness in children is caused by congenital conditions where they are born blind or arises from acquired conditions that occurred before the age of 5 years (Loulidi *et al*. 2025).

The major causes of blindness in children are congenital and developmental cataract, corneal scarring (from measles infection, vitamin A deficiency, and conjunctivitis of the newborn), retinal or congenital eye abnormalities, glaucoma, and retinopathy of prematurity (ROP). Approximately 40%–50% of these causes are preventable or treatable.

Good vision from early in life is essential for a child’s development. Normal vision in early childhood is necessary for a child’s vision to reach the full potential of normal adult vision. The earlier a child has vision problems and the more profound the reduced vision, the greater the negative impact on the development of their vision which is unlikely to ever reach normal adult vision. Therefore, any condition that deprives a child of vision from soon after birth can lead to developmental delay affecting motor, cognitive, social, and emotional development, which also impacts on their subsequent educational attainment (Sonksen and Dale 2002). Early identification and management of eye conditions are, therefore, essential if affected children are to reach their full potential in many areas of their lives.

In 1995 the World Health Organization (WHO) and United Nations International Children's Emergency Fund (UNICEF) launched the Integrated Management of Newborn and Childhood Illness (IMNCI) strategy to promote integrated services to reduce mortality and morbidity among young children from the main treatable and preventable diseases in countries with high under five mortality rates (WHO 1997). IMNCI is an algorithm-based, symptom-led approach, where primary healthcare workers complete a structured assessment of the child which leads to classification of the condition and its severity and a specific management plan with guidance on how to counsel the mother. Over 100 countries have adopted IMNCI to varying degrees. Although IMNCI included ear conditions, eye conditions were not included, with the only mention of eye diseases initially being that ‘children with measles may develop conjunctivitis, which should be treated with tetracycline eye ointment’ and then later, when newborns were included, ocular prophylaxis for ophthalmia neonatorum (WHO 2019a, 2019b, 2014).

The WHO’s Prevention of Blindness Program first recommended integrating 13 targets for child eye care into child health policies in 2002 (WHO 2002) which apply to the Global South. The WHO then revised their postnatal care guidelines in 2022, and these now include newborn eye screening for the first time (WHO 2022). At a national level, the Tanzania Ministry of Health (MOH) included eye health in their IMNCI strategy in 2019 (Malik *et al.* 2020).

The first major global report to include eye care in child health was ‘Survive and Thrive: transforming care for every small and sick newborn’ (WHO 2019a, 2019b). The report highlights the importance of quality inpatient neonatal care and facilities for specialized and intensive newborn care, which is very costly, requiring infrastructure, electricity, good maternity care, and highly trained staff. The report states that screening and treatment for ROP should be standard care for newborns in tertiary-level facilities. ROP is an eye condition exclusively of premature, low-birthweight babies, with a higher risk associated with increasing prematurity, poor quality neonatal care, and other complications of preterm birth (Hong *et al.* 2022).

### IMNCI in Tanzania

Eye screening is not routinely conducted in most low-resource settings nor is eye health included in child health programmes or policies (Mafwiri *et al.* 2014, Burton *et al.* 2021). Recent studies show how eye screening can be included in child health programmes. (Malik *et al.* 2020, Mndeme *et al.* 2020).

During 2017–18 a pilot study with the Tanzanian MOH was conducted to develop and test an eye health module for inclusion in the IMNCI strategy (Malik *et al.* 2020). The eye health module aligns with the other modules, with an algorithm for eye conditions for children aged 0–5 years. The eye health module was endorsed in 2019 at a national IMNCI meeting and included in the national strategy and IMNCI programme. Since 2019 the MOH has trained >3 500 primary health care workers in IMNCI, including the eye health module.

The objectives of this study were to: (i) understand the barriers and enabling factors to the policy decision to include eye health in wider child health policies; (ii) understand how child eye health was adopted within national policies despite it not being part of the international guidance; and (iii) explore the influence of global factors on Tanzania’s national-level processes. The aim of the study was to learn lessons that can be used by other countries to adapt their IMNCI strategy to include eye health and for advocacy at a global level.

## Materials and methods

### Adaptation of the Shiffman and Smith health policy framework

The Shiffman and Smith framework was chosen to guide data collection and analysis as it has been used to explain the factors that influence policy change both nationally and internationally. It is relevant to this context as it enables us to examine the roles and influences of multiple actors in the policy process, and helps to clarify why some health issues are prioritized over others in agenda setting (Shiffman and Smith 2007, Appendix 1). The framework allows a structured, comprehensive critical analysis and comparability with health policy change in other settings (Tomlinson and Lund 2012, Shawar *et al.* 2015, Shiffman *et al.* 2016). The framework has been applied most prominently to maternal mortality and newborn survival but has also been used to indicate which factors have shaped priority for mental health and global surgery. Another advantage of the framework is that it has been used at both national and global levels in key health areas such as newborn health and so is applicable in the national context of this study while also exploring global influences (Shiffman 2014). The framework was chosen because of its comprehensive and structured approach to assessing the complexities of policy processes, and because it helps to break down and examine key factors influencing the policy, especially where multiple actors are involved in policy decisions in the context of a particular country.

The Shiffman and Smith framework was adapted for the current study by including elements of Walt and Gilson’s framework (Walt and Gilson 1994). This was to make it more broadly applicable to issues as well as agenda setting, which is specifically the focus of Shiffman and Smith. Certain factors were combined in subsections under their category heading, e.g. issue characteristics, for brevity. The inclusion of ‘political feasibility’ and ‘recent shifts and changes in policy’ was taken from Walt and Gilson’s framework (Walt and Gilson 1994). The extent to which national-level policy decisions are influenced by global-level policy and the role of local determinants in decision making and specific eye health issues were also included (Table 1).

**Table 1.** Adapted Shiffman and Smith framework

Element	Description	Factors shaping political priority
Actor power within eye and child health community at national and global level	The strength of the different sets of individuals and organizations concerned with the issue	Policy community cohesion: strong national community cohesion backed by shared local research enabled rapid integration of eye care into IMNCI Leadership: joint leadership through national and international academics widely respected by decision makers Guiding institutions: organisations or co-ordinating mechanisms with a mandate to lead the initiative (not seen in Tanzania) Civil society mobilization: the extent to which grassroots organizations (or NGOs based on grassroots) have mobilized the necessary support from international and national political authorities (not applicable)
Ideas and influence of global ideas on national context	The ways in which the eye and child health communities understand and portray ideas	Internal frame: TOC previously developed by stakeholders in which there was agreement on problem and solutions, with one solution being the inclusion of eye care in IMNCI External frame: global influence of presentation of eye health being part of child development and the move from ‘survive’ to ‘thrive’ in child health agenda Presentation of eye health as ‘missing’ from IMNCI and a gap in child health resonated strongly as external frame
Political contexts global and national	The environments in which actors operate	Policy windows: IMNCI national review timing (unknown in advance to policy community) allowed for ratification of inclusion of eye health within national policy (policy window) Benefits from global shift in child health agenda from ‘survival’ to ‘thrive’ and supporting early childhood development (recent shifts and changes) Global governance structure: child and eye health institutions providing a platform for effective collective action (not applicable) Political feasibility: politically feasible due to decision making at national level and cost implications being included within IMNCI programme (political feasibility) Recent shifts and changes in policy: recent changes in structure of strategy allowing greater flexibility and feasibility to include eye health (recent shifts and changes)
Issue characteristics at the national level	Features of the problem	Credible indicators: paucity of data in low-resource settings but local evidence of burden and solutions which was presented to MOH within existing child health framework Severity: life-long effects of blindness on child and family and implications for communities and societies Effective interventions: availability of new low-cost technologies enabled policy makers to include in existing programmes

### Key informant interviews

Initial interview topic guides were based on the adapted framework (Table 1). Key actors were invited for in-depth semi-structured interviews. This method of enquiry was chosen as it allows exploration of concepts and ideas that emerge and evolve over the course of the interviews, while maintaining a comparable structure for each interview. The semi-structured topic guide ensured that we covered key factors based on our framework but was open-ended enough to allow unexpected issues to emerge (see Topic guide in the online Supplementary material). Flexibility in the questions asked was deemed important, as it was anticipated that key topics would be raised that were not included in the framework. The topic guides could therefore be adapted in later interviews to take account of issues raised in earlier interviews.

Interviews were conducted in person for all participants based in Tanzania, in a location of their choice, which included their workplaces, the office of the Co-principle investigator, or elsewhere. The in-person interviews were conducted in June 2022 and were led by the lead author with a co-author taking notes. Other participants, who were located in multiple countries, were interviewed online by the lead author over a period of 3 months from July to September 2021. The interviews, which lasted 45–60 min, were all undertaken in English and were audio recorded.

The interviews were transcribed and detailed notes were also taken during the interviews. Transcriptions were made by a professional transcriber and checked for accuracy by the lead author. The interview data were anonymized to protect confidentiality. Anonymized qualitative data were securely stored on encrypted laptops and in the author’s institution data repository for the standard period of 10 years.

### Sampling of key actors

The key actors were selected based on their influence and involvement in (i) the development of the eye module in the IMNCI programme in Tanzania, (ii) eye or child health in Tanzania, and (iii) global-level policy involvement in child or newborn health that could have influenced Tanzanian policy change.

Purposive sampling was used because many of the key actors were identified through their involvement in the policy-making process and policy documents. Snowball sampling was also used during interviewing, and participants were specifically asked if there were other actors who would be relevant to interview who were then invited to participate. The use of both purposive and snowball sampling allowed data saturation to be reached, when all major themes had been identified and additional interviews were unlikely to reveal new information.

The key actors were identified in several ways: through the author’s knowledge of Tanzanian health policy actors; identification from policy documents, in particular the joint WHO/UNICEF report ‘Survive and Thrive: transforming care for every small and sick newborn’; and asking known child-health leaders about who they believed to be key actors. Actors were purposively chosen from key organizations such as the MOH (maternal and child health and eye health departments), international bodies (WHO, UNICEF), national experts in child eye health or IMNCI, non-governmental organisations (NGOs) supporting eye care in Tanzania, funders, and the managing editors, first authors and content leads of the WHO/UNICEF report in order to ensure a good representation of views (WHO 2019a, 2019b). Five participants suggested by the managing editors and first authors of the joint WHO/UNICEF report were also interviewed. One funding agency representative was invited but did not respond. No other individuals declined to be interviewed and no interviews were repeated.

A total of 31 interviews were conducted with 14 actors based in Tanzania and 17 based in different countries. A summary of the organizations represented, the roles of those interviewed, and their gender are shown in Table 2. The NGO representatives included were those who were already actively involved in eye health and child health in the country. These were international eye NGOs with local offices and their representatives.

**Table 2.** Key actors interviewed

Key actor category	Institution/department/role	Gender	Current base country
MOH officials (3)	Maternal and child health Eye health	Male 2 Female 1	Tanzania (3)
International bodies (8)	World Health Organization UNICEF	Female 5 Male 3	Tanzania (2) USA (4) Switzerland (2)
Academia/technical experts (11)	Ophthalmologists, paediatricians, IMNCI national facilitators Universities/academics	Female 9 Male 2	Tanzania (5) USA, Canada, Australia (3) UK (3)
National representatives of NGOs who support eye care (7)	Five international NGOs working within Tanzania	Female 6 Male 1	Tanzania (4) USA (3)
Key funding agencies (2)	Two global funding agencies	Female 2 Male 0	USA (2)

### Data analysis

A data-extraction template was designed, based on the Shiffman and Smith framework adapted during the process of the data collection (Table 1). Interviews with the global health actors were analysed to understand the global policy context and what effect, if any, the global-level context had on the policy change in Tanzania.

The data-extraction template was adapted as required during the interview process and during analysis, but the initial codes were based on the framework determinants.

Each participant’s data was summarised using the framework elements as initial codes, ‘working down’ to form a coding matrix with the adapted framework factors used to form second-level sub codes. Data were analysed iteratively as soon as possible after the interview. As interviews progressed, earlier interview transcripts and notes were reviewed and were reflected in an adapted topic guide with new questions to test emerging themes. New data were then compared with the codes and categories, and developing themes were tested during subsequent interviews. This process was continuous throughout the interviews.

At the end of the interviews the main themes were defined and refined using all the interview data and then tested using multiple sources. For example, different interest groups (MOH officials, funders, health professionals) were compared, as were different written sources of information. Any contrasting views and ‘outliers’ were actively sought and reviewed. The lead author conducted all the coding. The Consolidated Criteria for Reporting Qualitative Research was used to ensure comprehensive planning of the studies. The subsequent reporting took into consideration the three domains of research team reflexivity (personal and relationship with participants), study design (theoretical framework, participant selection, setting, data collection), and analysis and findings (data analysis and reporting) (Tong *et al.* 2007). Specifically we used domain 1 to reflect and write the paragraph on reflexivity. Domains 2 and 3 were used to plan the methodology of the study and then to write it up.

### Reflexivity

Two co-authors are paediatric ophthalmologists and researchers in Tanzanian child eye health and both were involved in the pilot study to test an eye module for inclusion in the IMNCI programme in Tanzania (Malik *et al.* 2020). The evidence generated contributed to the MOH decision to include eye health in the IMNCI national strategy, and so both were key actors in the IMNCI policy making process in Tanzania. This had important implications for data collection, analysis, and interpretation of results. Both researchers had intimate knowledge of many of the events and knew some of the participants, and hence there is a risk of bias from assumptions from knowledge of previous research and knowing the key actors and a focus on eye health actors so that those who work in different sectors are not as well represented. However, the advantage was a greater awareness of the context and the history of events, which added depth to the analysis. Study participants who were not known to the researchers were aware of their credentials, experience, and the institutions they represented. It is, therefore, likely that the data collected were influenced by the participants knowing the interviewers, which may have led them to withhold information that was commonly known and to be more positive (or not divulging anything that could be construed negatively towards the researchers) in their responses.

Ethical Approval was obtained from the authors’ institutions. Consent was obtained from all participants before data collection.

## Results

This study identified the key determinants of policy change to integrate child eye health into the national policy in Tanzania, summarised in Table 1. The detailed results are presented below.

### ‘Actor power’ within the eye and child health community at national and global levels

#### Policy community cohesion

Although participants reported strong international community cohesion, this factor did not influence the integration of eye care into newborn care policy in Tanzania. From the interviews it became clear that there was a small group of key actors in child eye health in Tanzania who both knew each other well and were well-connected to policy makers. This is not unexpected from such a highly specialized area of health care. Hence, it was clear that NGOs and technical experts who were advocates for eye health had a high degree of cohesion on issues and worked together to advocate for child eye health. As an NGO interviewee stated:

We have been working on this issue for a long time and know we are a small community, so it is important for us to support each other (Participant 1, NGO)

This cohesion meant that when the opportunity to include eye health in IMNCI arose there was already strong cohesion on other aspects of child eye health, and as one technical expert put it:

There is no good reason why this [eye health] should not be included [in IMNCI] (Participant 1, technical expert)

The IMNCI official from the MOH also noted:

The agreement between the [technical experts] was clear (Participant 1, MOH)

Thus, the cohesion was noted from those external to the child eye health community which contributed to the actor’s power to influence the MOH.

There was also community cohesion amongst the global actors regarding the inclusion of eye care in newborn health guidelines, but only specifically for ROP services and not any other eye health issues, from academics to funders and NGOs. The policy community in newborn health was described by one of the NGO actors as:

Quite a small group working on newborn [care] and… those relationships… go back at least a decade, I think (Participant 2, NGO)

It was, therefore, a close-knit community and many of the issues of newborn health, including eye health, had been discussed over many years in technical sessions and discussions, as explained by this funder:

Those of us in a global public health space were pretty much at the same knowledge level around things like preventing child blindness, ROP, oxygen use… [I] just learned an extraordinary amount through the various technical sessions and discussions (Participant 1, funder)

Therefore, the issue of including eye health benefitted from having a relatively small and well-formed group who had collaborated for a decade on newborn health. Newborn health was considered for many years a neglected area of child health and therefore a small group of advocates including both academics and those working in the NGO sector worked together to develop and present the evidence for its priority. This led to a very cohesive group that had been advocating for newborn health, and when evidence for eye health in newborns and premature babies was present they were able to unite quickly to include eye health. The policy level discussions on ROP at an international level were separate from the policy community in Tanzania and there was no crossover as there was no engagement with the Tanzanian actors and communication of the agenda. Therefore, the local policy community in Tanzania remained distinct and separate and was able to lead on its own national priorities.

#### Leadership

Leadership in this case was provided by an academic collaboration between two universities, one national and one international. The MOH official noticed that this collaboration was:

Clearly … capable of leading [the initiative] (Participant 2, MOH)

The interviews suggested that these institutions were respected by the other key actors and NGO community and one NGO representative commented:

We had worked with them before and it has been a fruitful partnership (Participant 3, NGO)

As well as noting:

It is important to have their technical expertise (Participant 2, NGO)

This respect of the technical expertise and previous collaborations meant that the leadership was effective in bringing together all the key actors to focus on the issue.

The global child health community, outside Tanzania, also benefitted from leadership from academics and technical experts who were advocating and providing advice in multilateral organizations, as noted by one NGO representative:

So there are some people who are very strong in this community. So, advocates at WHO, UNICEF, also, voices like … have been incredibly powerful in this community in pushing the agenda forward …and really good at making sure that the things get done and that the pieces of research are out there… (Participant 4, NGO)

Although this leadership at the global level on ROP did not directly influence the specific issue in Tanzania (which was IMNCI and not ROP), the leadership could, however, have contributed by providing a favourable policy environment and an awareness of certain actors in the global picture.

### ‘Ideas’ and influence of global ideas in national context

#### Internal frame

The internal frame and the agreement of the policy community on the causes of child eye problems were high and there was an openness to solutions. Many actors referred to the long-standing relationships among the child eye health community in Tanzania, who had a history of collaboration on funded projects. An example mentioned by one of the NGOs was a project between the NGO and the two academic institutions on primary eye care for children, which had taken place 5 years previously, undertaken by two of the co-authors. This was described by the NGO representative as:

‘Very helpful for us to understand this problem in Tanzania’ and that it ‘really spurred so many of us on to take action’ (Participant 5, NGO)

During this project a theory of change (TOC) was developed collaboratively which was described as:

Such an important process (Participant 2, Development partner)

We have used that [theory of change] for many funding applications after (Participant 6, NGO)

It made it easier for us to understand the issues and explain it to others with a local context (Participant 7, NGO)

Although the TOC provided a common understanding of the causes of the problem of blinding eye diseases in children, the solutions were less clear. Some actors from the MOH admitted during interviews that they had not heard of IMNCI before the first stakeholder meeting at the start of the project but found it to be a ‘common sense’ approach and ‘very practical’. Another participant from an NGO saying, ‘we must try every approach and do everything we can that could work’. The key actors, therefore, felt able to support the inclusion of eye care in the IMNCI strategy as a potential solution.

The global actors focussed specifically on ROP within newborn health and considered it a quality-of-care issue, as noted by one academic:

[ROP is a] quality improvement issue … it’s like a ‘signal issue’ … if they can’t manage that [ROP] well, then they probably can’t manage all the other critical care issues that will arise (Participant 3, Technical expert)

Awareness of the impact of poor-quality neonatal care on the eye was a very powerful internal frame used by global actors.

#### External frame

The external frame focussed on eye health being ‘missing’ from IMNCI. When the solution of including eye care in IMNCI was advocated to the MOH they were also able to see:

‘All the experts and organizations—local and international—seem to be in agreement’ which ‘made us feel we will be able to make this [the eye IMNCI strategy] work well’ (Participant 3, MOH)

One powerful strength noted in the interviews from this external frame was that including child eye health became an issue for MOH policy makers and the broader policy community, adding a sense of ownership. This was reflected in interviews with MOH representatives with their repeated use of the word ‘we’ when discussing the eye IMNCI strategy:

We could see that this idea [including eye health in IMNCI] was something that made sense for all of us, why would we not do it? (Participant 2, MOH)

This sense of ownership by the MOH became apparent after the first stakeholder meeting of the research project, as the MOH led the development of the eye module. The MOH used their own team and processes and took responsibility for the production of the module, stating:

We felt we could manage this well within our department and it was our responsibility for our people to make sure it was done in the proper manner (Participant 1, MOH)

The eye module was initially developed in Swahili (Tanzania’s national language) with technical assistance from an academic collaborator and was then translated into English. The MOH used their own team and processes and took responsibility for producing the module, which was critical in their ownership of the programme and influential for the policy change.

The lack of eye health in the IMNCI global strategy was noted at initial stakeholder meetings which included international experts and funders. The international experts influenced the policy change through their evidence and agenda to include child eye health in child health policies, rather than retaining global strategies or policies which were found to be lacking, in particular the IMNCI strategy. The global policies were found to be rather disjointed and without clear enough details, however the overall direction of inclusion of eye health and the ‘whole child’ gave credence to the direction the key actors decided to take to go forward with including eye health.

There was no global strategy on eye health, and this was noted by international bodies and the MOH:

Highlighting the lack of inclusion of eye health in child health strategies was revealing and led to a lot of engaged discussions as to why and what we (in the local context) could do about it (Participant 4, technical expert)

There were stakeholder meetings where the academics highlighted the lack of global guidelines which made all the key actors aware:

We can see that this (eye health) is vital and necessary, but it has not been given the leadership at that global level (Participant 2, MOH)

The evidence the research team in Tanzania presented made the benefit of early detection clear, which influenced the MoH and international funding bodies that Tanzania could be a test case and they were prepared to include eye health in the IMNCI national programme:

It is clear that the global-level strategy is lagging behind… but we can lead the way here (locally in Tanzania) (Participant 4, development partner)

### ‘Political contexts’ global and national

#### Policy windows

Unknown to the child eye health advocates in Tanzania, an IMNCI strategy review by the MOH and key stakeholders, including WHO African region (AFRO), was planned for May 2019. The meeting took place shortly after the eye health module had been pilot tested. This meant that the inclusion of eye health in the IMNCI strategy could be ratified and included in national policy very quickly. The MOH interviewee said:

The timing [of the IMNCI pilot study] was perfect (Participant 3, MOH)

The WHO representative also said:

The timing could not have been better [for the development of the eye module] (Participant 5, development partner)

The timing of the development of the eye module was immediately before the internal review at WHO’s national and regional offices, which made it possible for it to be reviewed and included. A WHO AFRO interviewee described the regional and global shift in WHO strategic thinking on how IMNCI could move beyond child survival to support the child ‘thrive’ agenda.

The global policy window was occasioned by the shift of the global child health agenda from ‘survive’ to ‘thrive’, which provided an opportunity to advocate for the importance of including eye health. There was also a general move towards more comprehensive care for the newborn, as noted by one global-level NGO interviewee:

Making sure that it’s comprehensive care, that you’re not just trying to focus on one thing. So that might be why [eye health is gaining prominence]; it might just be part of the overall trend. (Participant 6, NGO)

#### Political feasibility, culture, and recent shifts and changes

The MOH in Tanzania assigned a specific person to lead the IMNCI strategy, which indicated that it was given some political priority. One of the characteristics of the IMNCI strategy is that although it is globally led, there is scope for national adaptation, and new modules can be introduced without affecting the rest of the strategy. The MOH interviewees explained that there was a precedent to adapt the strategy nationally:

We added ‘well child’ and ‘severe bacterial infections’ at the last [IMNCI] review (Participant 2, MOH)

Although these modules had been guided by global leverage, the experience meant the policy makers were open to adaptation at a national level.

MOH interviewees also explained that in-service training of the primary health care workers in IMNCI had changed from an 11-day face-to-face training programme to distance learning with 3 face-to-face training days over a 3-month period. The MOH representative indicated that this had practical and economic implications:

It was not possible before to add more as the training was already too long and expensive… But now this training is very flexible (Participant 1, MOH)

The change to distance learning drastically reduced costs (by 70%), reducing the burden on health facilities as staff were not required to be absent for 11 days, and allowed the MOH greater flexibility to make changes to the training programme.

At the global level there is a move in the childhood agenda to focus on more than ‘survival’ issues, meaning that child development and reducing morbidity and disability also require consideration. A global-level academic reflected on this, saying:

So not just the survive but the thrive—and as soon as you move into the thrive you have to be much more intentional on every aspect of the baby, not just their eyes alone but eyes and brain, and growth, and bonding, and support, and support to families and so on (Participant 6, technical expert)

### ‘Issue characteristics’

#### Credible indicators, severity, and effective interventions

From a public health perspective, the eye health of children has several challenges compared with other child health issues. Firstly, the outcome of most interventions, visual acuity, is not measurable in infants and is difficult to measure in pre-school-age children. In addition, due to visual development and neuroplasticity the final acuity outcome needs to be measured at the age of ≥ 5 years. Secondly, visually impairing conditions are not as common as other childhood conditions and there is a paucity of prevalence and incidence data, which hinders advocacy efforts. (Burton *et al.* 2021) Thirdly, while there are effective methods for child eye screening, they are not as easy to administer as screening methods for other childhood conditions and are often viewed as ‘too specialized’ for routine child healthcare workers. (Malik *et al.* 2018)

One advantage in the Tanzania setting was the presence of local evidence of the burden of child eye problems and potential solutions from recent studies (Mwende *et al.* 2005, Mafwiri *et al.* 2014). Studies had also shown that training primary health workers in child eye health was effective at detecting eye conditions at the primary care level (Malik *et al.* 2020, Mndeme *et al.* 2020). The MOH representative explained that this local evidence was vital for their decision to include the eye health module:

The evidence was clear that this was an issue we had to address for the children in Tanzania, and that we could (Participant 1, MOH)

We have local evidence of what is the problem and what can be the solutions in Tanzania (Participant 2, MOH)

We thought it may be difficult to include eye health but after seeing it has been implemented in parts of the country, we thought we can do this everywhere (Participant 3, MOH)

Another important factor highlighted was the availability of a simple, low-cost ophthalmoscope, called the ‘Arclight’. It is an alternative to a device called the direct ophthalmoscope and can be used to examine the front and back of eyes for any eye diseases including cataract and retinoblastoma. This technology has been tested and validated in Tanzania and other low-resource settings, providing evidence that it can be effective (Mndeme *et al.* 2020). Testing children’s eyes with the Arclight was included in the eye health module and its importance was noted by the MOH and WHO:

This Arclight is really useful as we can give one to every primary healthcare worker (Participant 2, MOH)

Now we have something [the Arclight] that they [primary health workers] can use which makes [eye examination] possible (Participant 8, development partner)

### Challenges in the policy making process

A key challenge was the tension that was raised regarding the implications of the policy for (i) increasing expectations and workloads for the primary health workers and (ii) costs of devices and further training. As noted by the IMNCI national facilitators:

The only issue is that the primary health care workers are already doing so much and whether they can do more (Participant 10, IMNCI national facilitator)

However, another facilitator noted:

This is something so essential that I know they (the primary healthcare workers) are capable (Participant 11, IMNCI national facilitator)

Some of those working at the NGO level and key funding agencies were also concerned about ‘overloading’ the primary health care workers. Many of these concerns were debated and addressed by the evidence both locally and internationally. Local and international experts who had conducted research in Tanzania were able to show that this could work in this context:

It was so helpful to be able to present the evidence from Tanzania showing both quantitatively and qualitatively that primary health care workers could be trained to check young children’s eyes quickly and easily (Participant 4, technical expert)

This presentation of the evidence was a collaboration between international and national participants. Thus the issue of whether it was suitable for primary health care workers was addressed by local evidence.

Similarly, the costs of the training and devices was raised by the key funding agencies. However, interestingly, the MOH representatives were able to see the cost-effectiveness:

The device is by far the lowest cost for screening we have seen and for what it does (Participant 1, MOH)

Although there was debate surrounding the costs, overall, the cost-effectiveness of the device ($12), which was an additional cost to an existing programme, was seen to be more affordable. In addition the time taken to examine using the device once trained has been found to be very quick (less than a minute) and therefore does not add significantly to the workload issue.

## Discussion

The key to successful policy change was good quality research within the local context, with all actors working together (cohesive policy community) including the decision makers (MOH actors). This collaborative approach coincided with the shift in the international agenda of moving from ‘survive to thrive’ in child health which was able to be used as leverage to include eye care in the national strategy.

The cohesiveness of the policy community was an important factor in influencing the inclusion of eye health in child health. Cohesion is important in effective advocacy, a factor that has been reported as missing in other areas of health, such as surgery and mental health (Tomlinson and Lund 2012, Shawar *et al.* 2015). The involvement of the MOH at an early stage in the research activities was influential as they were long-term collaborators in the research and became involved in the development and delivery of the eye care module (Malik *et al.* 2020), and hence were ready to include it when the policy window arose. This was similar to many other case studies, for example in Honduras where a highly effective working group formed with key stakeholders (MOH, United Nations Population Fund, United States Agency for International Development (USAID), UNICEF, other donors and agencies) produced a national plan to reduce maternal mortality (Solebo *et al.* 2017)

The importance of global networks has been highlighted in a policy framework designed to better understand their role in building political priority, drawing on examples from emergency medicine, maternal health, and musculoskeletal conditions (Smith and Rodriguez 2016, Shiffman 2018, Briggs *et al.* 2020, Chipendo *et al.* 2021). The global network was not directly useful in Tanzania as it was in the previous examples. Tanzania benefited from a national network which was made up of NGOs and technical experts. This was effective in framing the issue of including eye health by working in the policy environment with the MOH, which gave a sense of priority to the issue.

A study in three countries (Bolivia, Malawi, and Nepal) explored the determinants of newborn health priorities at a national level (Smith *et al.* 2014). Key findings were that the efficacy of solutions needed to be demonstrated in low-resource settings, and advocacy should build on existing national priorities, with a strong network of national and global leaders. Contextually relevant evidence may be particularly influential, as decision-makers want to invest in policy solutions that are likely to give results. This concurs with our experience in Tanzania which showed that the local evidence, also clearly relevant to the local context, was a clear deciding factor on proceeding with policy change.

A study from Argentina, which focussed on reducing blindness from ROP through policy change and implementation, also found a local policy community/network within a favourable regional/global policy environment to be important. Key factors were persistent advocacy by a group of national professionals, legislation initiated by a mother of an ROP blind child, and action by the Pan American Health Organization, which all set the agenda for policy change for the control of ROP in Argentina (Hariharan *et al.* 2018). The framing of eye health as ‘missing’ from IMNCI in Tanzania was also effective because the strategy was designed to be comprehensive. Local evidence of the size of the problem alongside a solution was compelling for policy makers.

In most countries health issues compete against one another for scarce funding. Certain issues can receive priority based on external funding by donors and NGOs which then influence national governments to prioritize those issues. One example has been HIV/AIDS which has received priority due to greater international funding, while other issues such as maternal mortality were side-lined for some time (Shiffman 2007). Improving neonatal outcomes is very costly, requiring infrastructure, electricity, good maternity care, and highly trained staff and has had issues with competing with funding. Integration of eye care within IMNCI or other child health programmes avoids the issue of competing with other child health issues, e.g. vaccinations or HIV/AIDs etc., at that primary care level, but does not address the whole patient pathway. Thus, donors such as USAID continue to fund the whole package of training of IMNCI which now includes eye care. This is a key factor not only in child eye health gaining priority but also in sustainability as a result of having allocated resources. However there remain challenges for those children who require treatment, despite some availability of government funding.

Witter *et al*. (2019) studied policy transfer and the use of evidence in eight low- and middle-income settings on a range of policies, including IMNCI. They structured their findings according to policy conceptualization, policy uptake or implementation, and further policy development. For conceptualization, the authors found that all reforms start with local recognition of a problem before being influenced in several ways. Ideas from other contexts are often brought by international agencies who partner with government. The policy change needs to be seen as meeting local needs and as being a policy developed within the country. The example of IMNCI in Nepal followed this route of substantial within-country conceptualization with bilateral and multilateral partner support in policy transfer. This example could occur in other settings as IMNCI benefits from regional and national reviews, which are an opportunity to exchange ideas between countries, and from being a strategy that is designed to be flexible and adaptable. If local needs of child eye health are established, then external partners can support the concept of including eye health in IMNCI.

There can also be resistance to the acceptance or adoption of international ideas or rejection of advice from other countries. For example, Nepal resisted several WHO-recommended adjustments to clinical guidelines as they did not fit into their wider health system strategy or capacity (Witter *et al.* 2019). In Nepal, the drivers that led to guidelines being updated were from the local context and implementation issues. There was also greater credibility given to local evidence in Nepal, with globally published evidence mentioned less often. Therefore even if evidence is lacking for a policy, if it fits well in the local socio-political context, then it could still be taken up. Studies have shown that evidence used to inform policy is often not scientifically collected or applied, and it is important to highlight how evidence meets the socio-political concerns and priorities of the policy makers (Lavis *et al.* 2008, Witter *et al.* 2019).

At country level, policy makers and technical staff often use relationships with development partners for advice at all policy stages, and these personal relationships can be very important especially in smaller countries with long-term partners (Lavis *et al.* 2008, Koon *et al.* 2013, Cairney and Oliver 2017). International and regional meetings and technical assistance programmes can also influence learning, and within countries, pilot projects supported by international NGOs can play an important role in developing policies. In this study in Tanzania, the research study (Malik *et al.* 2020) using international partners played an important role in policy update which could be used in other countries for policy transfer.

The integration of eye care into IMNCI or other child health policies in other low- and middle-income settings may be achieved using the key factors for successful policy transfer to other settings by focussing on: local advocacy for recognition of the problem; the influence of partnerships with international agencies, as they have a role in influencing national policy in the absence of international policy; regional examples; and development of local evidence that is framed to fit with the local socio-political agenda. Leveraging existing small and cohesive policy communities and networks with wider interests (both eye and child health) is a key factor that can be used by child eye health advocates in other countries. Long-term multi-stakeholder collaborations were key and involving the MOH in previous research studies was vital to ensure they were on board from the start. The framing of child eye health as being necessary for comprehensive child health and/or child development with clear outlining of the problem and the solutions internally (within the policy community) and within the global policy agenda can be highly influential for policy makers. Policy makers could see that implementing the policy change was feasible, and it is vital that all the equipment necessary for the solution is affordable and locally available, as was the case in Tanzania. A critical determinant in the Tanzanian context was local evidence of the severity of child eye health problems, with evidence of effective interventions and credible indicators for child eye health. These factors highlight the importance of building a local research agenda.

### Limitations of the research

A limitation is that all the interviews were analysed by one researcher (A.N.J.M.), which increases the possibility of bias from subjectivity. This was counteracted by using the Shiffman and Smith framework both in data collection and analysis as this more structured approach can reduce the subjectivity, as well as improve comparability.

Another limitation is that the main author (A.N.J.M.) was an actor in this study. This had important implications for data collection, analysis, and interpretation of results. The author’s knowledge of the events and some of the actors increases the risk of bias from assumptions on who was a key actor and who was not. However, the advantage of this was a more thorough awareness of the context of the policy change and history, which can add greater depth to the quality of the analysis. The interpretation of the data will also have been influenced by the author’s prior knowledge of the issues and interest in child eye health, e.g. on policies, where the policies were lacking, and the challenges faced by children and their families.

There are a number of challenges for the implementation of screening for eye diseases in children in Tanzania that were not addressed in this research but are critical. Screening is one step in the patient pathway to treatment and, while critical, there are important barriers with the referral to care, transport costs and availability, and receiving adequate care and its costs. While there are tertiary eye centres in Tanzania that can provide all the relevant eye care treatment these are concentrated in specific areas and for many living in Tanzania extremely far away. However one of the key issues in child eye health is late presentation due to the critical nature of timely treatment on the outcome, therefore screening is an essential first step. This then empowers the patient’s family to know there is something that can be addressed and also has implications for the wider society in advocacy for making treatment for these children available and accessible as they are no longer ‘invisible’. This also opens up the possibility for those who do not receive treatment, or receive it too late, to be supported with low vision. While this is not directly addressed in our research the policy change enacted can subsequently be used as leverage for further change.

### Implications for research

Policy research is needed focussed on the biases of policy makers, researchers, and medical professionals who find eye care difficult and challenging to implement, as well as on the stigma associated with blindness and low vision. Policy research at a global level on why child eye health has remained less visible within the newborn and child health agenda to date, and what key factors would generate greater global priority, are critical at this time due to the opportunities which have arisen due to expanding child health policies that now include ‘thrive’ and childhood development. The adapted Shiffman and Smith framework developed in this study can be used to explore agenda setting in other countries.

## Conclusions

As far as we are aware this is the first study of agenda setting in relation to eye health policy at national level with regard to child health policies. The adapted Shiffman and Smith framework used in this study was useful to draw out issues specifically for the inclusion of eye health in child health national programmes and may be used in other contexts to compare the experience to this study in Tanzania.

A global policy window has opened with a move in child health policy from the ‘survival’ focus to how a child can ‘thrive’ (WHO 2019a, 2019b). This window provides a major opportunity for the inclusion of eye health not only in IMNCI strategies but also in other national and global child health policies for young children. The lessons from the Tanzanian context can be used in other countries showing that it is possible for eye health to be given political priority by: (i) leveraging existing policy communities and networks; (ii) ensuring ideas are presented clearly within the context of current child health priorities internally and externally; and (iii) developing local evidence collaboratively with the policy community. To address the ‘gap’ governments, donors and advocates must collaborate at every stage from inception and evidence building to advocacy to ensure eye health becomes an integral component of IMNCI and other child health policies in other countries and at a global level. This policy work needs to be complemented by working on solutions to address treatment for children with eye problems and support for those with low vision. This is the first step in change to provide comprehensive eye care for children, no matter where they live.

## Supplementary Material

czaf029_Supplementary_Data

## Data Availability

The data underlying this article will be shared on reasonable request to the corresponding author.

## Reflexivity statement

This study reflects long-standing collaborations between the authors in research projects in Tanzania for over 5 years. The research reflects local priorities: the MOH recently included eye health into child health policy, and four of the co-authors have worked with the MOH Tanzania on child eye health for the last 5 years. The corresponding author is from a high-income country but has substantial professional experience in child eye health in Tanzania and this study was included as part of her PhD thesis. The research idea originated during the corresponding author’s PhD period and during work in Tanzania in collaboration with three of the co-authors. The co-authors span different levels of seniority and experience: among the co-authors there is a PhD student, and professors based both in Tanzania and the UK with experience in child health, eye health, epidemiology, as well as health policy expertise. The majority of the co-authors are women, two are people of colour, and one is from a low- and middle-income country where the study is based.

## Abbreviations

IMNCI: Integrated Management of Newborn and Childhood lllness
LSHTM: London School of Hygiene and Tropical Medicine
MOH: Ministry of Health
NGO: Non-governmental organization
ROP: Retinopathy of prematurity
TOC: Theory of change
WHO: World Health Organization
