# Supplementary material for: Integrating eye health into a child health policy in Tanzania: global and national influences
Source: Health Policy Plan. 2025 Jun 23;40(7):696–707. doi: 10.1093/heapol/czaf029 (PMC12360167; doi:10.1093/heapol/czaf029)
Supplement: czaf029_Supplementary_Data [file czaf029_supplementary_data.zip › CZAF029_Supplementary files/final revised HPP tables .docx]

**TABLES**

Table 1: Adapted Shiffman and Smith framework

| Element | Description | Factors shaping political priority |
| --- | --- | --- |
| Actor power within eye and child health community at national and global level | The strength of the different sets of individuals and organisations concerned with the issue | Policy community cohesion: Strong national community cohesion backed by shared local research enabled rapid integration of eye care into IMNCI  Leadership: Joint leadership through national and international academics widely respected by decision makers Guiding institutions: organisations or co-ordinating mechanisms with a mandate to lead the initiative (not seen in Tanzania) Civil society mobilisation: The extent to which grassroots organizations (or NGOs based on grassroots) have mobilized the necessary support from international and national political authorities (not applicable) |
| Ideas and influence of global ideas on national context | The ways in which the eye and child health communities understand and portray ideas | Internal frame: Theory of Change previously developed by stakeholders in which there was agreement on problem and solutions, with one solution being the inclusion of eye care in IMNCI External frame: Global influence of presentation of eye health being part of child development and the move from ‘survive’ to ‘thrive in child health agenda  Presentation of eye health as ‘missing’ from IMNCI and a gap of child health resonated strongly as external frame |
| Political contexts global and national | The environments in which actors operate | Policy windows: IMNCI national review timing (unknown in advance to policy community) allowed for ratification of inclusion of eye health within national policy (policy window)  Benefits from global shift in child health agenda from ‘survival’ to ‘thrive’ and supporting early childhood development (recent shifts and changes) Global governance structure: child and eye health institutions providing a platform for effective collective action (not applicable)  Political feasibility: Politically feasible due to decision making at national level and cost implications being included within IMNCI programme (political feasibility)  Recent shifts and changes in policy: Recent changes in structure of strategy allowing greater flexibility and feasibility to include eye health (recent shifts and changes) |
| Issue characteristics at the national level | Features of the problem | Credible indicators: Paucity of data in low resource settings but local evidence of burden and solutions which was presented to MoH within existing child health framework Severity: life long effects of blindness on child and family and implications for communities and societies Effective interventions: New low-cost technologies available enabled policy makers to include into existing programmes |

Table 2: Key actors interviewed

| **Key actor category** | **Institution/ department/role** | **Gender** | **Current base country** |
| --- | --- | --- | --- |
| Ministry of Health officials (3) | - Maternal and Child Health - Eye Health | Male 2  Female 1 | - Tanzania (3) |
| International bodies (8) | - World Health Organization - UNICEF | Female 5  Male 3 | - Tanzania (2) - USA (4) - Switzerland (2) |
| Academia/ Technical experts (11) | - Ophthalmologists, Paediatricians, IMNCI national facilitators - Universities/ academics | Female 9  Male 2 | - Tanzania (5) - USA, Canada, Australia (3) - UK (3) |
| National representatives of NGOs who support eye care (7) | - Five international NGOs working within Tanzania | Female 6  Male 1 | - Tanzania (4) - USA (3) |
| Key funding agencies (2) | - Two global funding agencies | Female 2  Male 0 | - USA (2) |
