# Supplementary material for: Integrating eye health into a child health policy in Tanzania: global and national influences
Source: Health Policy Plan. 2025 Jun 23;40(7):696–707. doi: 10.1093/heapol/czaf029 (PMC12360167; doi:10.1093/heapol/czaf029)
Supplement: czaf029_Supplementary_Data [file czaf029_supplementary_data.zip › CZAF029_Supplementary files/HPP Topic Guides .docx]

Topic Guides

**Topic guide 1: to explore key factors which led to the decision to include eye health in the IMNCI policy and programme in Tanzania**

Intro

- What organizations/networks do you belong to? Please describe your role within this

organization/network

- What in your opinion was the most important factor(s) facilitating eye care being included into IMNCI
- What were the largest barriers

Ideas/issue characteristics

- How would you define child eye health including:
  - the main issues in child eye health
- What do you understand to be the size of the child eye health problems in Tanzania
  - how does this compare to other child health/ other health issues
  - any measures/data you know about child eye health in Tanzania
- What do you consider to be the main solutions to improve child eye health (at primary care level) including:
  - any specific interventions and their effectiveness/cost effectiveness
- How important would you consider child eye health in comparison to other child health issues
- What level of evidence are you aware of for eye care interventions for children
- How has the case been made for including eye health into IMNCI

Actor power

- Who do you consider the key individuals in child health policy making in Tanzania
- Who would be considered leaders in the eye health/ child health/ child eye health communities/policy makers in Tanzania
- Which are key organisations in eye health/ child health/ child eye health communities/policy making
- Is there any grassroots organisations involved in advocacy of child eye health
  - If so, how have they been involved
- Do you think all those involved (above) work together collaboratively?
  - Were there any issues where all agreed
  - Were there any issues where there was any disagreements or tensions
- Who are the key individuals involved in IMNCI in Tanzania

Political context

- Can you think of any significant events or developments that have helped to draw attention to the child eye health in Tanzania?
  - Can you think of any events or developments that have detracted the attention and any missed opportunities?
- Does child eye health align with child health strategies in Tanzania
  - If so, how?
  - what have been/ are the main child health strategies
- What factors about IMNCI influenced the ease of/hindered inclusion of eye health
  - how is IMNCI implemented in Tanzania
  - is the training managed locally/ nationally
  - are there other examples of how IMNCI in Tanzania has been adapted and differs from the global policy
- How is IMNCI delivered in Tanzania
  - What has been adapted/adopted compared to the international programme
  - what key changes have occurred at the international level in IMNCI over recent years

Tanzania health system factors

- What is the structure of primary health care/ child health care in Tanzania
- Who are the PHWS and what level of eye health training would they have in the routine educations and training
- What are the current responsibilities on PHWs
- Where does the funding for PHC/ IMNCI/ child health in Tanzania come from

Tanzania historical perspective

- What in your opinion have been the most important eye health initiatives, and specifically in child eye health in Tanzania over the last 20 years (prompt: specific examples if none given by interviewee)
- Which key actors have worked together to improve eye health/child eye health previously
- What components of primary eye care and specifically child eye health were already in place in Tanzania

Integration

- What are other key child health issues which have been integrated in recent years in Tanzania
- How else has policy on IMNCI changed since first started in Tanzania

Policy transfer/ uptake/ adaptation/ evidence use

- Are there other examples of health initiatives that Tanzania has adapted and integrated into global health policies/programmes

Conclusion

- Anything else?
- Who else would you recommend I interview
